# Supplementary material for: Phosphoproteomic Profiling Reveals mTOR Signaling in Sustaining Macrophage Phagocytosis of Cancer Cells
Source: Cancers (Basel). 2024 Dec 19;16(24):4238. doi: 10.3390/cancers16244238 (PMC11674635; doi:10.3390/cancers16244238)
Supplement: Supplementary file 1 [file cancers-16-04238-s001.zip › cancers-3360540-supplementary.pdf]

Supplementary Materials for

Phosphoproteomic Profiling Reveals mTOR Signaling in Sustaining Macrophage Phagocytosis of Cancer Cells

Bixin Wang [1,†], Xu Cao [1,†], Krystine Garcia-Mansfield [2,3,‡], Jingkai Zhou [1,‡], Antigoni Manousopoulou [1], Patrick Pirrotte [2,3], Yingyu Wang [4,*], Leo D. Wang [1,5,*], Mingye Feng [1,*]

* Corresponding authors:

Yingyu Wang, Email: yingyuwang.yw@gmail.com

Leo D. Wang, Email: lewang@coh.org

Mingye Feng, Email: mfeng@coh.org

Figs. S1 to S4

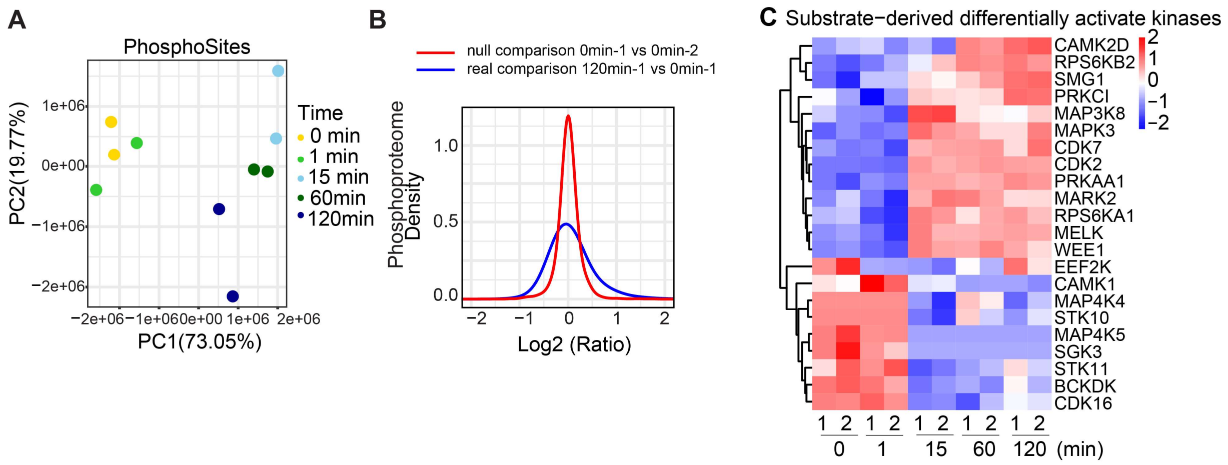

Supplemental Figure S1. A multiplexed quantitative analysis of the phosphoproteome was applied to analyzing phagocytosis of cancer cells by macrophages.

(A) Principal component analysis showing unsupervised clustering of all phosphosites from macrophage co-culture at each of the 5 time points.

(B) Log2 (ratio) distributions of representative null and real comparisons for phosphoproteome.

(C) Heatmap showing unsupervised clustering of differentially active kinases, detected via time series analysis using linear model through limma. Clustering of phosphosites was performed using Pearson's correlation coefficient, with samples ordered by time point from left to right. Kinase activities were scaled across the rows and represented as z-scores by color.

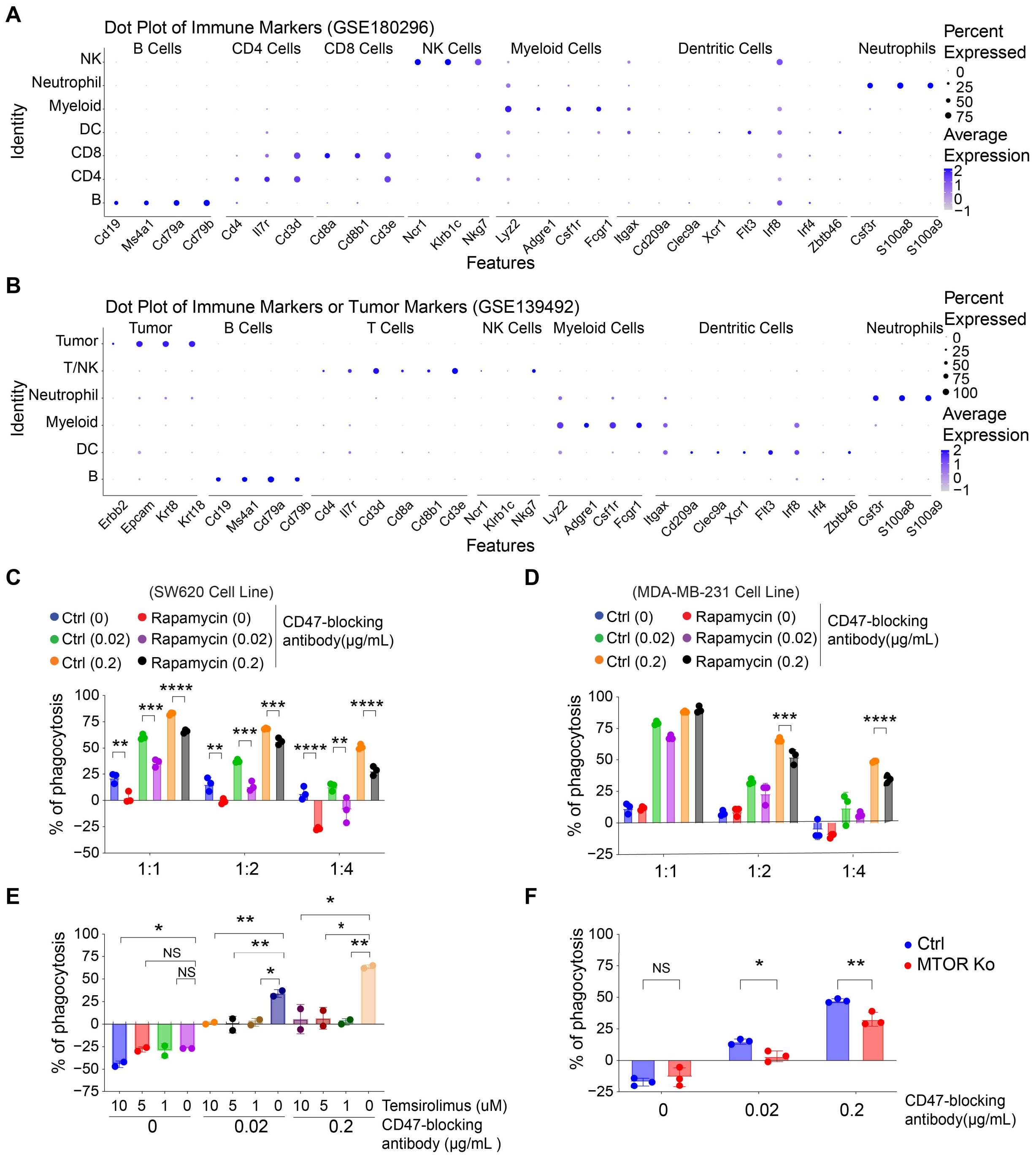

Supplemental Figure S2. Phosphoproteome profiling identified the activation of the mTOR pathway during macrophage phagocytosis.

(A) Dot plots of immune cell marker of GSE180296: B cells (Cd19, Ms4a1, Cd79a, Cd79b); CD4 T cells (Cd4, Il7r, Cd3d); CD8 T cells (Cd8a, Cd8b1, Cd3e); NK cells (Ncr1, Klrb1c, Nkg7); Myeloid

cells (Lyz2, Adgre1, Csf1r, Fcgr1); DCs (Itgax, CD209a, Clec9a, Xcr1, Flt3, Irf8, Irf4, Zbtb46); Neutrophils (Csf3r, S100a8, S100a9).

(**B**) Dot plots of immune cell marker and tumor marker of GSE139492: Tumor cells (Erbb2, Epcam, Krt8, Krt18); B cells (Cd19, Ms4a1, Cd79a, Cd79b); T cells (Cd4, Il7r, Cd3d, Cd8a, Cd8b1, Cd3e); NK cells (Ncr1, Klrb1c, Nkg7); Myeloid cells (Lyz2, Adgre1, Csf1r, Fcgr1); DCs (Itgax, CD209a, Clec9a, Xcr1, Flt3, Irf8, Irf4, Zbtb46); Neutrophils (Csf3r, S100a8, S100a9).

(C) Rapamycin pretreatment (100nM) significantly inhibits phagocytosis of SW620 colon cancer cells by BMDMs at different E:T ratios. N = 3.

(D) Rapamycin pretreatment (100nM) significantly inhibits phagocytosis of MDA-MB-231 breast cancer cells by BMDMs at different E:T ratios. N = 3.

(E) Temsirolimus pretreatment at different concentrations significantly inhibited phagocytosis of Raji cells by BMDMs. N = 2.

(F) Phagocytosis assay using BMDMs with control knockdown or MTOR knockdown, co-cultured with Raji cells. N=3.

Data represented as means ± SD. NS indicates not statistically significant; *p < 0.05, **p < 0.01, ***p < 0.001, ****p < 0.0001, as determined by one-way ANOVA (E) and Two-way ANOVA (C-D and F).

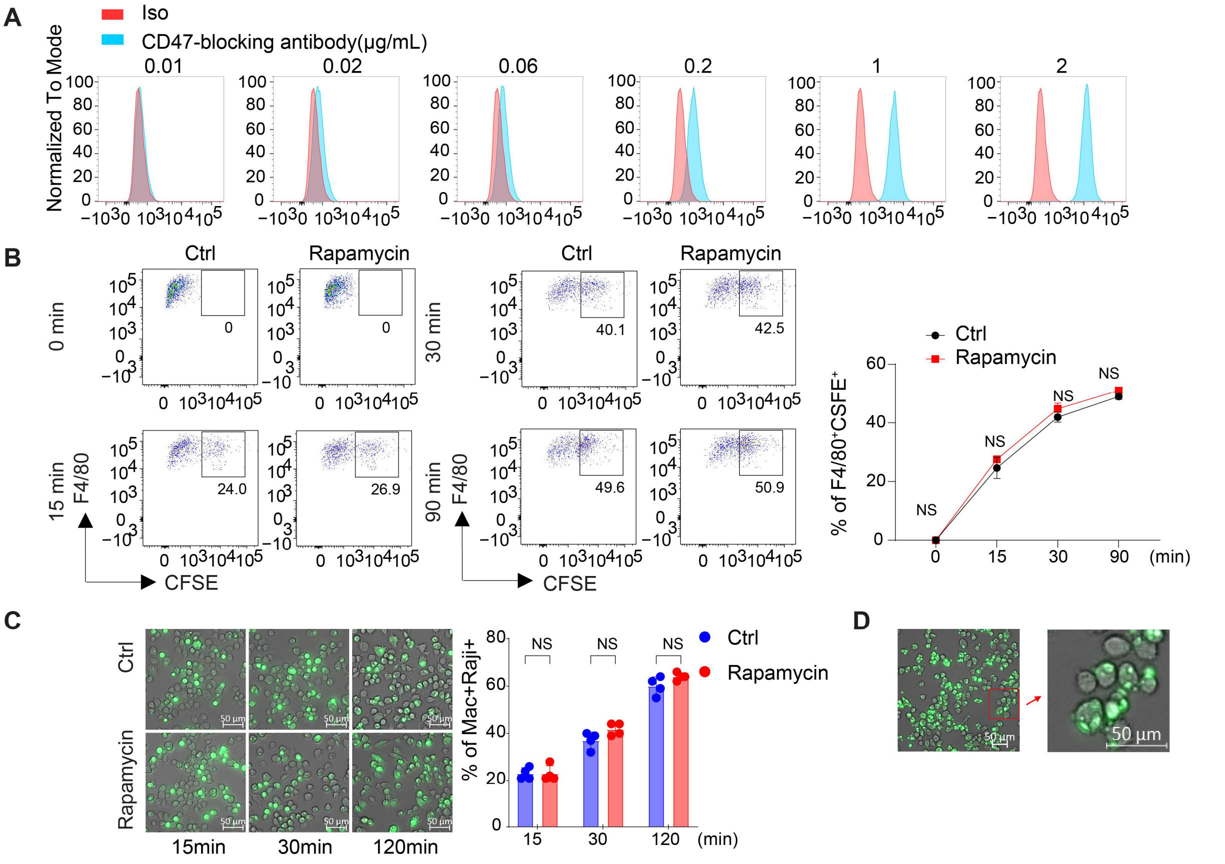

Supplemental Figure S3. Blockade of mTOR signaling impairs the capacity of macrophages for phagocytosis but not their affinity with it.

(A) Histograms showing the detection of CD47 expression on Raji cell by various concentrations of the CD47-blocking antibody (0.01, 0.02, 0.06, 0.2, 1, 2 μg/mL), along with a summary of CD47 MFI at different concentration of antibodies. N=3.

(B) A flow cytometry-based phagocytosis assay. CFSE-labeled Raji cells were incubated with BMDMs treated with control vehicle or rapamycin in the presence of 2 μg/mL of CD47-blocking antibody. Representative FACS plots showing the expression of F4/80$^+$CFSE$^+$ cell at 15min, 30min, and 90min of the control group and rapamycin group. The percentages of F4/80$^+$CFSE$^+$ cells at different time points for both groups were summarized. N = 3.

(C) A microscopy-based phagocytosis assay. CFSE-labeled Raji cells were incubated with BMDMs treated with control vehicle or rapamycin in the presence of 2 μg/mL of CD47-blocking antibody. Representative images (left: acquired using 20x/0.5 objective and a 1.6x optovar) and quantification (right) are shown. N =4.

(D) Representative microscopy image (acquired using 20x/0.5 objective) of macrophages continuously phagocytosing multiple target cancer cells, with a zoomed-in inset highlighting the phagocytic details. N=4.

Data represented as means ± SD. NS indicates not statistically significant; $^{*}p < 0.05$, $^{**}p < 0.01$, $^{***}p < 0.001$, $^{****}p < 0.0001$, as determined by Two-way ANOVA (B-C).

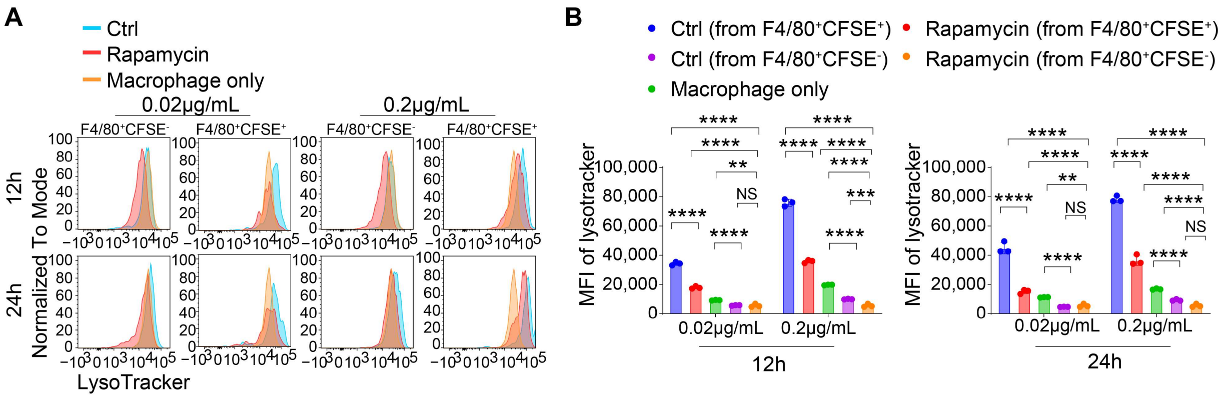

Supplemental Figure S4. Blockade of mTOR signaling delays phagosome maturation.

(A) Supplementary data of the experiment shown in Figure 5A-B. Representative FACS plots displaying the intensity of LysoTracker staining from F4/80$^+$CFSE$^+$ and F4/80$^+$CFSE$^-$ BMDMs in the presence of 0.02 or 0.2 μg/mL of CD47-blocking antibody at 12h and 24h. Data were collected for three groups: control macrophage + Raji, rapamycin-pretreated macrophage + Raji, and macrophage-only. N = 3.

(B) Summary of the Mean Fluorescence Intensity (MFI) of LysoTracker staining in (A). N = 3. Data represented as means ± SD. NS indicates not statistically significant; *p < 0.05, **p < 0.01, ***p < 0.001, ****p < 0.0001, as determined by two-way ANOVA (B).

00D7
